# A Nurse-Led Multimedia Intervention to Increase Patient Participation in Recovery After Knee Arthroplasty: Hybrid Type II Implementation Study

**DOI:** 10.2196/36959

**Published:** 2022-05-19

**Authors:** Jo McDonall, Bernice Redley, Patricia Livingston, Ana Hutchinson, Richard de Steiger, Mari Botti

**Affiliations:** 1 School of Nursing and Midwifery Faculty of Health Deakin University Geelong Australia; 2 Centre for Quality and Patient Safety Research-Monash Health Partnership Deakin University Geelong Australia; 3 Centre for Quality and Patient Safety Research-Epworth HealthCare Partnership Geelong Australia; 4 Epworth HealthCare Epworth Victor Smorgon Chair of Surgery Department of Surgery Richmond Australia; 5 The University of Melbourne Melbourne Australia

**Keywords:** patient participation, multimedia, nurse-facilitated, knee arthroplasty, orthopedic surgery, acute care, nurse, participatory medicine, digital technology

## Abstract

**Background:**

Advances in digital technology and the use of multimedia platforms to deliver information provide clinicians with a unique opportunity to develop innovative ways to consistently provide high-quality, accessible, and evidence-based information to support patient participation. Introducing new technologies into everyday acute care clinical practice can be difficult.

**Objective:**

The aim of this paper was to provide a description of an implementation strategy and the subsequent evaluation undertaken to examine the contextual factors important to the successful adoption of new technology by nurses in the context of acute postoperative care.

**Methods:**

Implementation of the intervention and process evaluation was undertaken in 3 phases: phase 1, preimplementation stakeholder engagement and identification of barriers and enablers to implementation; phase 2, supported implementation of the intervention; and phase 3, evaluation of uptake, usability, and acceptability of the intervention in clinical practice.

**Results:**

The outcomes of the implementation of the multimedia intervention in the context of acute postoperative care were positive. Of the 104 patients in the intervention group, 103 (99%) received the intervention. All 103 patients completed the 8-item intervention questionnaire and 93.3% (97/103) were interviewed on day 3 to evaluate usability, uptake, and acceptability. Of these 97 patients, almost all (n=94, 91%) found the program easy to use and most (n=64, 62%) could view the MyStay Total Knee Replacement program as often as they wanted. The findings also suggest that the time to implement the program was minimal (5-10 minutes). Collaboration with nurses and patients before and during implementation to identify potential barriers to successful implementation of the intervention was essential to develop timely strategies to overcome these barriers. To ensure end-user engagement, careful consideration was given to nurses’ views on who was responsible for facilitating this intervention.

**Conclusions:**

The findings provide evidence that the structured implementation of the multimedia intervention was robust and successful in terms of patient participant recruitment and application; however, it was difficult to assess the level of engagement by nurse clinicians with the program.

**Trial Registration:**

Australian New Zealand Clinical Trials Registry (ANZCTR) ACTRN12614000340639; https://anzctr.org.au/Trial/Registration/TrialReview.aspx?ACTRN=12614000340639

## Introduction

### Background

Advances in digital technology and the use of multimedia platforms to deliver information provide clinicians with a unique opportunity to develop innovative ways to consistently provide high-quality, accessible, and evidence-based information to support patient participation. To date, multimedia tools have been used in a wide range of health situations, including providing preoperative education to prepare patients to undergo specific procedures and provide consent [1-7]; assisting patients to make informed decisions regarding treatment [8,9]; enabling self-management in chronic illness [10]; supporting postoperative care (eg, how to use a patient-controlled analgesic pump after surgery) [11]; and improving patient overall satisfaction [12]. There is emerging use of technology to facilitate patient participation in acute postoperative contexts. The effectiveness of using digital, multimedia platforms to enhance patient participation in their care is directly affected by nurses’ attitudes [13]. If nurses perceive that a new technology will be burdensome, unreliable, or does not fit into their workflow, they are less likely to promote its use by their patients. Implementation strategies that specifically target the range of individuals involved in delivering patient care and organizational processes are needed to successfully introduce and embed novel technologies and interventions into clinical practice [14,15].

**Figure 1:**
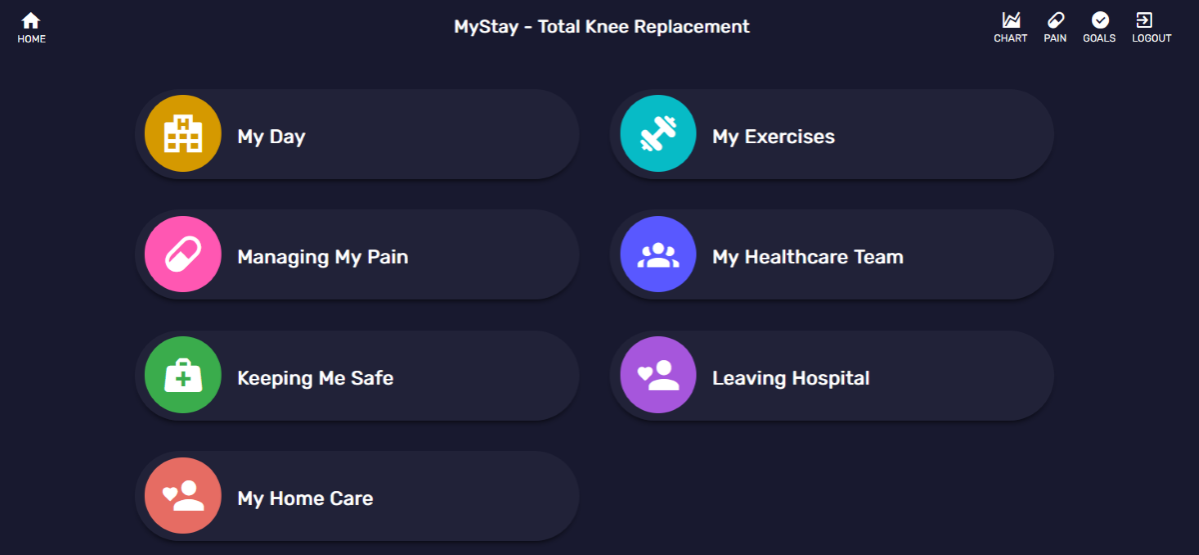
MyStay Total Knee Replacement landing page examples.

**Figure 2:**
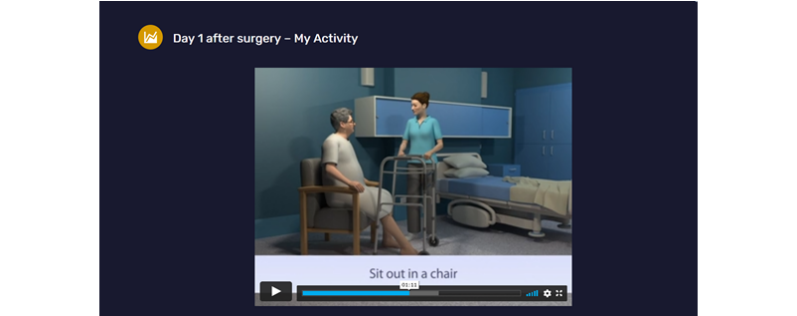
MyStay day 1 after surgery My Activity page.

**Figure 3:**
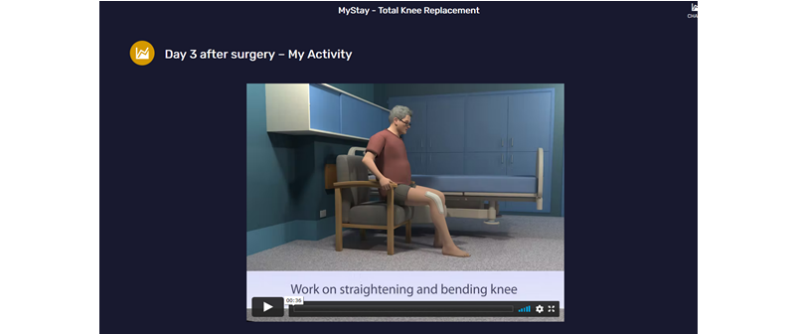
MyStay day 3 after surgery My Activity page.

A novel multimedia intervention, *MyStay Total Knee Replacement* (*MyStay TKR),* was developed specifically for use by patients after undergoing total knee replacement by Enlighten Health, a medical multimedia production company specializing in validated content for patient and clinical education. *MyStay* *TKR* was developed using an iterative, multi-method approach aimed at ensuring that program content was valid and reflected an optimal balance among procedure-specific best evidence, current clinical practice, and patient preferences. *MyStay TKR* was designed to be both nurse-facilitated and patient self-directed; that is, accessed and used independently by patients as a stand-alone program packaged for iPad (Apple Inc) presentation [16]. The intervention has two interacting components: (1) information tailored to each day of recovery to enhance patients’ understanding of their goals of recovery and (2) explicit information outlining how to achieve their recovery goals. Multimedia through iPad technology was selected as the intervention most likely to be effective in influencing patient participation in the context of acute postoperative recovery because it places minimal burden on nurses and patients, has continuous availability, and is intuitive and easy to use [17]. The multimedia intervention was designed to deliver information that was explicit, actionable, nonambiguous, and tailored specifically to the daily goals that support patient recovery after total knee replacement surgery (Figures 1-3).

### Objectives

This paper reports an implementation strategy and evaluation undertaken to examine the contextual factors important to the successful adoption of *MyStay TKR* by nurses and patients in the context of acute postoperative care. A cluster randomized controlled trial was conducted to test the effectiveness of the intervention and is reported elsewhere [16]. The implementation strategy and evaluation reported here were designed to assess the degree to which each element of the program was implemented. The specific objectives of this evaluation were to determine the following:

1. The system or environmental factors that may have had an impact on the implementation of the intervention.
2. Patient usability, uptake, and acceptability of the multimedia intervention in the context of acute recovery after surgery.

## Methods

### Implementation and Process Evaluation

A prospective concurrent implementation evaluation of the *MyStay TKR* intervention was undertaken. The intervention was implemented using a structured standardized approach with boundaries established to limit variation [18]. Consistent evidence-based implementation processes were used on the wards and involved multiple methods [19]. The implementation and process evaluation were undertaken in 3 phases: phase 1, preimplementation stakeholder engagement and identification of barriers and enablers to implementation; phase 2, supported implementation of the *MyStay TKR* intervention; and phase 3, evaluation of patient usability, uptake, and acceptability of the *MyStay TKR* multimedia tool in clinical practice. The implementation strategies used and data collected to measure the effectiveness of the implementation process in each phase of the study are summarized in Table 1.

**Table 1:** Methods and data collection across 3 stages of the MyStay Total Knee Replacement (MyStay TKR) trial implementation.

Trial phase		Implementation strategies used	Evaluation methods
Phase 1: preimplementation (nurses)		Purposive group interview Ward meetings and in-service education Flyers and handouts Email correspondence	Analysis of interview and meeting data
Phase 2: implementation (nurses and patients)		Daily ward visits (intervention and control wards) Daily field observations One-to-one and ward meetings Handouts and flyers Correspondence through patients’ bedside whiteboards	Analysis of meeting notes and observation field notes using qualitative content analysis
**Phase 3: evaluation**			
	**Nurses**		
		Observations of clinical practice and incidental staff feedback	Analysis of field notes
	**Patients**		
		Uptake and use of the MyStay TKR intervention by patients Patient self-reported questionnaires	Analysis of interview data and descriptive statistics using SPSS software (version 23; IBM Corp)

### Phase 1: Stakeholder Engagement

#### Leadership Engagement

Before implementation of the *MyStay TKR* intervention, nurses (n=4) were purposively sampled to participate in a group interview. All 4 participants were permanent staff employed on the orthopedic ward and included 1 (25%) educator, 2 (50%) senior registered nurses, and 1 (25%) graduate nurse. The focus of the discussion was how best to embed the multimedia intervention into nurses’ everyday practice and to identify strategies to mitigate nurses’ perceived barriers to successful implementation.

#### Staff Education Sessions

A range of activities were undertaken before the commencement of the trial to support successful implementation of the *MyStay TKR* intervention; these included informing all surgeons, nurses, and physiotherapists about the study and expectations of their involvement, as well as ensuring that the clinicians were exposed to the intervention and familiar with content and navigating the program on the iPads. At ward and in-service meetings, the project was described in detail along with a demonstration of the animation intervention designed for patients and any questions were addressed. In total, 3 formal ward meetings were conducted before implementing the program on each ward, and additional in-service meetings were held on each ward until >80% of the ward nursing staff had received training in using the *MyStay TKR* tools. In addition to the daily meetings, 1 *night* meeting was held on each ward to ensure that the permanent night staff were also well informed about the study. Orthopedic surgeons and physiotherapists were also involved in information sessions related to the study to ensure their support for the study.

#### Development of Promotional Materials

Handouts and flyers were developed and placed at the nurses’ stations and in break rooms to engage nurses and inform them of the study. On each flyer the researchers’ contact details were provided to invite questions or suggestions. Nurse unit managers, physiotherapists, and ward nursing staff were sent regular emails to provide updates on the stages of the study throughout the trial period.

### Phase 1: Data Collection

The semistructured interview with key clinical nurse leaders was recorded and transcribed for later analysis to identify barriers and facilitators to implementation of the *MyStay TKR* intervention. During the staff education sessions, field notes captured reports of barriers and the suggested strategies identified during each of these encounters.

### Phase 2: Supported Implementation of the MyStay TKR Intervention

#### Overview

Application of the intervention procedure involved a structured process that included promotion and awareness raising, patient engagement, and development of tailored solutions to implementation barriers. These processes are outlined in Table 2.

**Table 2:** Application of the intervention procedure.

Key process	Procedure
Identification of patients enrolled in the trial	At the beginning of each day shift, nurse unit managers, and associate nurse unit managers were informed of the following: The researcher presence on the ward A list of patients enrolled in the trial on their ward identifying the “day” after surgery The exact number of iPads required per ward per day and ensure that they were charged and ready for use
Application of intervention procedure	Identify the nurse responsible for the care of patient participants Confirm with the nurse that the patient is enrolled in the study and will need to view the iPad animation Identify day 1 patients and provide and secure the iPad and explain how to use the device and navigate the program Patients instructed to watch the animation on the iPad and call their nurse once they have finished to discuss the content The nurse will confirm and clarify any questions the patients may have regarding the information The iPads remain with the patient for the duration of their stay Laminated flyer attached to the patients’ medical record and note on the individual patients’ communication board were used to remind clinical staff that the patient was in the study Patients’ nurses are responsible for ensuring that the iPad is charged overnight Telephone call to the wards nightly at 10 to remind them to charge the iPads
Strategies used throughout the trial to maintain engagement by nursing staff	One-to-one discussions among ward nurses, physiotherapists, surgeons, and the nurse researcher Telephone calls to associate nurse unit managers on afternoon shifts at 8 PM each day to ask that they remind staff to charge the iPads overnight Regular attendance at ward meetings by the nurse researcher where questions could be answered and strategies discussed to assist with the implementation Laminated cards were placed in patient notes, and a sign was placed on the whiteboard above the patient bed area Patients themselves reminded staff to attend to the iPad; for example, to plug in the iPad for charging overnight

#### Promotion and Awareness Raising

To support successful implementation of the *MyStay TKR* intervention, the researcher (JM) conducted daily ward visits for the duration of the trial. The intent of these visits was to promote uptake of the *MyStay TKR* intervention and to support adherence to key processes of care. At these ward visits, the researcher ensured that any casual staff were familiar with the trial, placed flyers in patients’ notes and on bedside whiteboards to alert clinical staff that the patient was enrolled in the trial, obtained ongoing feedback from the health care team about any barriers to implementation of the intervention, and observed practices related to implementation and usability of the intervention (patient and clinician engagement).

#### Patient Engagement and Facilitation of Patient Participation

During the implementation phase, patients together with the researcher navigated the *MyStay TKR* animation on the iPad. Each section of the program was explained until patients were comfortable with access and could follow the program. This introduction to the program took 5 to 10 minutes, depending on the patient’s familiarity with the iPad. The iPad was then left with the patients, who were informed that they could use the program as often as they wished. Patients were also instructed to call their nurse to inform them that they had finished watching the program. The nurse would then clarify any questions the patient may have regarding the information provided, and it was anticipated that a discussion regarding the goals of the day would ensue. Physiotherapists are crucial in mobilizing patients after surgery and restoring mobility in the knee joint. As such, physiotherapists were involved in the development of *MyStay TKR* content through the Delphi process and reviewing of the exercise components of the multimedia, as well as subsequently during implementation of *MyStay TKR.* During implementation, physiotherapists encouraged patients to view the *MyStay TKR* modules on the iPads. This was achieved during their initial visit on day 1 with patients and also throughout the patients’ stay during follow-up visits. Physiotherapists asked patients to watch the exercise component of *MyStay TKR* in their absence.

#### Tailored Solutions

During the implementation phase, the researcher obtained ongoing feedback from staff and patients regarding barriers and facilitators to implementation and use of the *MyStay TKR* intervention. To support successful implementation of *MyStay TKR*, the researcher worked with the nursing team based on the ward to identify time-sensitive solutions to these barriers.

### Phase 3: Usability, Uptake, and Acceptability of the MyStay TKR Intervention

The effectiveness of the implementation strategy was evaluated using data on the uptake and use of the *MyStay TKR* by patients as well as by obtaining patient feedback about the uptake, usability, and acceptability of using the program to support their recovery. All patients who were randomized to an intervention ward were given the *MyStay TKR* evaluation questionnaire, an 8-item self-report tool specific to the intervention. This questionnaire was designed to uncover the ease of use, satisfaction with, and effectiveness of the multimedia program to aid in the patients’ recovery.

### Data Analysis

The Theoretical Domains Framework was used to inform the identification of barriers and facilitators to practice and behavior change with regard to both clinicians and patients [20].

There were three components to the analysis of the study data:

1. Qualitative content analysis was performed to identify key themes that emerged from the focus group discussion and the staff education sessions. The transcripts were independently reviewed for factual content by 2 researchers (JM and MB), who formed agreement on key emergent codes for thematic analysis. The codes were then grouped to identify key themes and subthemes. All members of the research team reviewed the identified themes and subthemes, and the thematic structure was determined by consensus.
2. Throughout the implementation phase the researcher collected field notes describing these communications, and any observations made by the researcher related to implementation of the intervention were transcribed in a field diary. These notes were coded for recurring themes in terms of barriers and facilitators.
3. Descriptive statistics were used to analyze the uptake and use of *MyStay TKR* as well as patient responses to the *MyStay TKR* evaluation questionnaire.

### Ethics Approval

The cluster randomized controlled trial and the implementation evaluation were approved by the health service and university institutional ethics committees (Epworth HealthCare Human Research and Ethics Committee, 598-13, and Deakin University Human Research Ethics Committee, 2013-195).

## Results

The study results are described in 3 sections to reflect each phase of the implementation evaluation.

### Outcomes of Phase 1

#### Overview

In total, 3 themes were derived from the analysis of the transcripts of the clinician group interview, ward meetings, and one-to-one communications with nurses, and these were used to inform how to embed the intervention into everyday practice on the wards. The themes were as follows: (1) the potential burden of introducing the intervention for staff, (2) perceived difficulties associated with the age of patients and ease of use of technology, and (3) concerns about safety and security of the iPad within the ward (Table 3). There were no concerns raised by physiotherapists or surgeons regarding application of the intervention in the preimplementation stage.

**Table 3:** Perceived barriers to implementation and strategies used to address these barriers.

Barrier or concerns identified by nurses	Illustrative quote	Strategies used to address concerns or barriers
Potential burden of introducing the intervention for nursing staff	“Can you guarantee this [iPad intervention] will not increase our already busy workload? I mean, if we have to spend time going through this iPad [intervention] then it’s going to make it harder for us isn’t it...I mean, we just don’t have the time.” [Nurse ID 2] “I don’t know, I think there’s a lot going on in the morning...we [the nurses] are busy and flat out. First thing it is probably easier if someone else does it [goes through the program with the patient] and not leave it up to the nurses?” [Nurse ID 1]	Implementation of the intervention on day 1 of patients’ recovery was carried out by the researcher to ensure that patients could use the iPad and navigate the program Patients who were classified as postoperative day 1 received an explanation of the iPad and navigation after handover and before breakfast, at approximately 8 AM each day
The age of patients and ease of use of technology	“With the older patients we may have to teach them how to use the iPad [intervention] or they may not be able to use it at all. Do you think this is very realistic, I mean for them to use it?” [Nurse ID 3] “Yes, some of them have other comorbidities, you know, such as arthritis, it may be harder for them...we will have to push it for them? If that’s the case, I don’t think we will have the time.” [Nurse ID 4] “I don’t think it should be an issue, my grandparents have one and they use it ok.” [Nurse ID 1]	A flyer to assist patients to navigate the program themselves was provided to all patients Once patients were familiar with the iPad the nurses felt that they were able to focus on the content of the program
Security and safety of the equipment and infection control	“So where are you going to put it [iPad intervention]? You don’t want it to get in the way. There’s not much room anyway with all their [patients] stuff. Perhaps it could be put on the bedside tables so we can get it out of the way if we need to?...What about keeping it clean, what do you think?...Have you thought about the cross contamination?” [Nurse ID 1] “Yes, you have to make sure it doesn’t walk either...if it’s not secure, things walk here, how will you make sure it stays with the patient? And what about if it gets dropped they are very sensitive these iPads...what will happen there...do you have lots of replacements?” [Nurse ID 3]	To address security concerns, the iPad was secured to each patient’s movable bedside table with a locked cable Each iPad was secured inside a locked tough case that was drop-, smash-, and splash-proof The infection control nurse approved the cleaning protocol for each iPad before transfer to another patient. Wiping the iPad and all associated material (cords, case, etc) with an alcohol-impregnated cloth was approved as sufficient cleaning between patients Cleaning occurred on collection of the iPad when a participating patient was discharged from hospital

#### Potential Burden of Introducing the Intervention for Nursing Staff

Nurses expressed concern that they may need to facilitate the use of the iPad and assist patients to navigate the program the first time they were exposed to *MyStay* TKR. They thought that this would take a significant amount of time, particularly during the busy morning period that includes clinical handover and patient assessment. There was also worry that there may be an additional burden on nursing staff during the patients’ stay where they may have to reintroduce and reiterate aspects of the *MyStay TKR* content with patients each day, thus increasing their workload.

#### The Age of Patients and Ease of Use of Technology

There were mixed attitudes regarding the age of the patients and their ability (physical and mental) to use the iPad. Some (2/4, 50%) of the nurses indicated that older patients may be unfamiliar with portable devices or unable to use them.

#### Safety and Storage of Equipment

Nurses were worried about the physical location of the iPad in patients’ rooms and stated that the device could add to existing clutter and be removed or stolen or dropped and broken. The potential for cross-contamination and risk of infection was also raised.

### Outcomes of Phase 2

#### Overview

Table 4 outlines the patient participant characteristics at baseline. During the implementation phase, the strategies outlined in Table 3 were applied to address the potential barriers to successful implementation.

**Table 4:** Patient participant baseline characteristics (N=104).

Characteristics		Values
Age (years), mean (SD)		65.25 (9.77)
**Sex, n (%)**		
	Male	40 (38.5)
	Female	64 (61.5)
**Living arrangements, n (%)**		
	Living communally	88 (84.6)
	Living alone	16 (15.4)
**Marital status, n (%)**		
	Partnered	84 (80.8)
	Not partnered	10 (9.6)
	Widowed	10 (9.6)
**Country of birth, n (%)**		
	Australia	76 (73.1)
	United Kingdom	11 (10.6)
	Other	8 (7.7)
	Europe	6 (5.8)
	Asia	2 (1.9)
	New Zealand	1 (0.9)
**Language spoken at home (primary), n (%)**		
	English	102 (98)
	Italian	1 (0.9)
	Other	1 (0.9)
**Employment status (preadmission), n (%)**		
	Retired	52 (50)
	Full time	24 (23.1)
	Part time or casual	16 (15.4)
	Unemployed	7 (6.7)
	Other	5 (4.8)

Of the 104 participants recruited for the intervention group, only 1 (0.9%) patient was unable to receive the multimedia intervention in the trial. This deviation was due to factors outside the control of the study: the patient had a serious postoperative complication and therefore was unable to receive the intervention. In total, 94.2% (97/103) of the patient participants were interviewed on day 3. Reasons for participants not interviewed were as follows: too unwell, not available on the ward at the time, or declined to be interviewed. Interview duration ranged from 12 to 75 minutes. Most (94/97, 97%) of the interviews were conducted between 9 AM and 2 PM at the patients’ bedside; the rest (3/97, 3%) of the interviews were conducted at a later time (after 5 PM) at the patients’ request.

During the interviews, patients reported a range of structural, clinician-related, and patient-related barriers to use of the *MyStay TKR* program. These barriers were addressed as they were identified as described in the following sections.

#### Structural Factors

The physical location of the iPads presented a problem when trying to ensure that the program was always available for patients when they wanted to access it. Because of physical constraints of space, several options were tested until agreement was reached about the ideal location. Initially, the iPads were secured to the patients’ bedside trolleys to enable the iPad to be moved around if patients decided to sit out of bed; however, this caused problems for the food services staff who found it difficult to find room to place patients’ food trays. The decision to move the iPads to the patient’s bedside locker was made in consultation with the patients as well as food services and nursing staff. The cord that tethered the iPad to the bedside table was long enough for the iPad to be placed on the bed should patients decide to sit out of bed and view the presentations. On several occasions nurses and the services staff moved the patients’ iPads to the back wall “to keep it out of the way.” This then prevented patients from watching the iPad as they could not reach it. These prohibitive behaviors were fed back to the nurses caring for the patient on the day.

#### Clinician-Related Factors

Nurses’ attitudes toward the program were critical to its successful implementation. In the third week after commencement of the project, 3% (3/97) of the patients commented that nurses (n=2) had stated that they were “sick of these iPads” and “these iPads just get in the way.” These comments can influence patients to question the use of the program and can negatively affect their confidence to ask nurses questions related to the program. To address these issues, group and one-to-one discussions were held with the nursing staff to determine what strategies might be implemented to overcome these perceptions. Field notes revealed that of the 103 iPads used over a period of 14 months, there were 17 (16.5%) with flat batteries; however, the majority (n=13, 76.5%) of these were in the initial rollout phase. Reasons for the flat batteries outlined by nursing staff were “forgot to put on charge,” “no charger available,” “needed the charging plug for another appliance,” and “unable to charge” (2/103, 1.9% of iPads were *missing* the charging adapter). Throughout the trial period this practice improved, with only 3.9% (4/103) of the iPads noted with flat batteries after approximately 1 month following commencement of the trial.

#### Patient-Related Factors

Difficulties encountered by patients in using the iPad included being unable to watch the entire program because of sleepiness or tiredness, difficulty remembering to watch the program, and being too unwell to watch because of pain or other complications. Strategies were discussed with each patient and their nurse during the daily visit, and methods to overcome barriers to use were agreed. For example, the patients who were too tired to watch all of the program at once were directed to watch only small clips at a time and nurses would remind them to watch more throughout the day. If patients were in pain, they were reminded by nurses to watch the program later in the day. No barriers were identified by patients in relation to the information delivery using the iPad.

### Outcomes of Phase 3: Uptake, Usability, and Acceptability of the MyStay TKR Intervention

Of the 104 recruited participants, only 1 (0.9%) randomized patient was unable to receive the multimedia intervention in the trial because they experienced a serious postoperative complication (cerebrovascular accident) and were therefore unable to receive the intervention.

All 103 patients completed the 8-item intervention questionnaire on day 3. Overall, 66% (68/103) of the patients reported that they had viewed the iPad program more than once in the previous 24 hours, 29.1% (30/103) had viewed the program once, and 4.8% (5/103) reported that they had not viewed the program in the previous 24 hours. Reasons for not viewing the iPad program were as follows: watched the entire program on days 1 and 2 after the surgery, unable to view because of illness, too tired to watch at the time, and they planned to watch the program later in the day.

Almost all (94/103, 91.3%) patients found the program easy to use. In total, 62.1% (64/103) of the patients reported that they felt they could view the program as often as they wanted. Reasons for not viewing the program as often as they would have liked included feeling too tired or too unwell (24/33, 73%), technical issues with the iPad having a flat battery (11/33, 33%), and concerns about the voiceover on the program disturbing patients in shared rooms (1/33, 3%; Table 5).

**Table 5:** Patients’ reasons for not viewing the program on the iPad as often as they wanted (some patients indicated multiple reasons; N=39, 38%).

Reason stated for not viewing the program as often as wanted	Values, n (%)
Too tired (including visitors)	12 (36)
Too unwell (predominately nausea)	12 (36)
iPad did not work properly when I had the opportunity to watch (battery flat)	11 (33)
No time, (patient) too busy	7 (21)
Pain too severe	6 (18)
iPad not available when I had the opportunity (not in reach)	4 (12)
Forgot about watching it	4 (12)
Did not understand the content	2 (6)

As the intervention was designed to be nurse-facilitated, patients were asked on day 3 whether the nurses responsible for their care had discussed the program with them in the previous 24 hours. Only 21.4% (22/103) of the patients reported that nurses had discussed the program with them in the previous 24 hours.

Patients’ reported satisfaction with the intervention was high, as reflected in a mean score of 8.63 (SD 2.05) out of 10. No problems with navigation of the program on the iPad were reported.

## Discussion

### Principal Findings

This evaluation of whether a multimedia intervention delivered through an iPad could be successfully implemented on acute orthopedic wards established that most (94/103, 91.3%) of the patients found the program easy to use, with their reported satisfaction with the intervention being high (mean score of 8.63, SD 2.05, out of 10), and the program required minimal time for orientation. Collaboration with clinicians and patients before and during implementation to identify potential barriers to successful implementation of the intervention was essential to develop timely strategies to overcome these barriers. To ensure end-user engagement, careful consideration was given to nurses’ views on who was responsible for facilitating this intervention. Several methods were adopted to ensure that nurses had the opportunity to discuss concerns and express their opinions about embedding the implementation into their everyday clinical practice. The effects on nursing staff workload, the physical location of the iPad, and the safety and security of the device were identified as key areas of concern and were addressed in the implementation plan.

The intervention was implemented using a structured, standardized, and evidence-based approach [18,19]. The intervention was designed to be delivered in the context of usual care delivery; however, nurses were reluctant to perform the initial orientation with patients because of concerns that instructing patients on the use and navigation of the iPads would be time consuming and would interfere with the provision of patient care.

On day 3 after the surgery, patients reported low levels of nurse engagement with the intervention. There are several possible explanations for this: it is possible that nurses were satisfied that patients were engaging sufficiently with the intervention or that there had been higher levels of interaction during the previous postoperative days. It is possible that nurses were not engaging with the *MyStay TKR* program and did not see it as a tool to set goals of care with patients to assist them with their recovery. The challenge for future studies is to demonstrate to nurses that these types of interventions will not have an impact on their workloads [18]. In fact, the time needed to explain the program to patients was very brief and could easily be incorporated into everyday clinical practice.

Embedding interventions into clinical practice has been reported to be challenging, particularly in the acute care setting where work is often fast paced, with nurses caring for patients who are acutely ill after surgery [20]. Implementation of the intervention in this study required nurses to facilitate interactions among themselves, the multimedia program, and patients to create opportunities for patients to discuss their goals of recovery and negotiate pain management. This element required a patient-centered approach [21-23], which can be difficult to achieve in practice when nurses perceive that their workload is already high. Several acute care studies have reported that nurses spend only a small amount of time with each patient [24-27], and the acuity levels of the patients in the postoperative context also result in some patients being allocated more time than others [28].

As with any new technology designed for patients in the clinical setting, ease of use is a primary design consideration. Most (64/103, 62.1%) of the patients reported that they were able to view the program as often as they liked without restriction. Patients also successfully navigated the program independently, and all (103/103, 100%) patients interacted with the program at least once a day. However, the patients’ acuity levels did limit their level of interaction. These findings are consistent with those of other studies that have evaluated the implementation of a multimedia intervention in acute care [17,29-31].

Reasons stated by patients for not interacting with the *MyStay* program were predominately related to the acuity of their illness rather than the program itself, suggesting that usability was not a problem. Consistent with the findings of Cook at al [29], the major barrier for patients in engaging with the *MyStay* program was tiredness and nausea, both common symptoms in the acute postoperative period. An advantage of the program being available 24 hours a day was that patients could access the program when it suited them. In previous studies where patients had limited access to interventions, usability was compromised [12]. A study by Chu et al [32] reported that 71% of patient time in hospital was considered *downtime*; that is, patients were not occupied with diagnostic tests or other activities. This suggests that there is ample opportunity for patients to engage with an intervention program throughout the day if there is flexibility in availability. An additional advantage of the multimedia platform is that patients’ families could also view these programs during their visits to help to reinforce the goals of recovery.

Nurses’ concerns that older age may hinder patients’ ability to use the iPad technology was not identified as a limiting factor in this study. Of the 103 patients, only 2 (2%) stated that they were *computer illiterate* and that this was a reason why the program was not easy to use. Advanced age was not identified as a factor affecting usability; indeed, a patient aged 95 years found the iPad so usable that he indicated he would purchase one when he was discharged. Our findings are similar to those of Cook et al [29] who found that patients can in fact interact with a multimedia device, regardless of age: 91.3% (94/103) of the patients reported it to be easy to use; reasons for the patients (9/103, 8.7%) who indicated difficulty included flat battery, lack of concentration because of health, or the sound was poor. The majority of these factors were rectified during the trial.

Creating an opportunity for patient participation without placing an additional burden on clinicians and patients was considered critical in this study. The *MyStay TKR* intervention was designed to be easily navigated by patients and nurses in the acute care environment [33]. Time spent by the researcher orientating patients to the technology was 5 to 10 minutes initially and then 2 to 5 minutes per day with individual patients. It is concluded therefore that the *MyStay TKR* intervention can be incorporated into everyday routine care, despite the acuity of the environment and the time required for nurses to allocate in applying (not facilitating) the program is low and feasible [32]. These findings are consistent with those of other studies that have implemented multimedia interventions for patients in hospital [17,34,35].

### Conclusions

Implementation of a nurse-led multimedia intervention to increase patient participation in recovery after total knee replacement was achievable. The findings demonstrated that the implementation of the *MyStay TKR* multimedia intervention was robust and structured and successful in terms of patient participant recruitment and application; however, it was difficult to assess the level of engagement by nurse clinicians with the program. Furthermore, the findings indicate that a multimedia program designed as a platform to promote patient participation within acute care environments that can present challenges to engagement is feasible and is associated with high patient satisfaction.

## Abbreviations

MyStay TKR: MyStay Total Knee Replacement

## Data Availability

Evaluation data are available upon request.
